# Rotational stability can be enhanced in revision anterior cruciate ligament reconstruction using the over-the-top augmentation technique compared to single bundle technique

**DOI:** 10.1186/s13102-023-00724-1

**Published:** 2023-09-15

**Authors:** Sumin Lim, Ki-Hoon Park, Do Young Park, Tae Hun Kim, Jeong-Hyun Koh, Jun Young Chung

**Affiliations:** 1 Department of Orthopedic Surgery, Ajou University School of Medicine, 164 Worldcup-ro, Yongtong-gu, Suwon, 16499 Korea; 2 Daprtment of Orthopedic Surgery, Armed Forces Yangju Medical Center, Yangju-si, Korea; 3 Cell Therapy Center, Ajou University Medical Center, Suwon, Korea

**Keywords:** Achilles tendon allograft, Anterior cruciate ligament reconstruction, Over-the-top augmentation, Revision surgery, Rotational stability

## Abstract

**Purpose:**

Revision anterior cruciate ligament (ACL) reconstruction is technically challenging due to mispositioned tunnels, bone loss, and tunnel enlargement, which may compromise graft fixation and result in failure. To obtain firm graft fixation and strength in one stage, we utilized an over-the-top augmentation technique using an Achilles tendon allograft in revision ACL reconstruction (OA-ACLR). This study compared OA-ACLR with single-bundle ACL reconstruction (SB-ACLR). We hypothesized that OA-ACLR would enhance the postoperative knee joint rotational stability.

**Methods:**

We retrospectively analyzed 47 patients who underwent revisional OA-ACLR and 48 who underwent primary SB-ACLR with minimum follow-up of 6 months. Knee instability was evaluated with the anterior drawer, Lachman, and pivot shift tests preoperatively and at the final follow-up. Side-to-side differences were compared with the non-affected side at the final follow-up. Function was evaluated using the IKDC subjective and Lysholm knee scores preoperatively and at the final follow-up.

**Results:**

The groups did not differ in terms of sex, age, BMI, and etiology. There were no significant differences in concomitant surgical procedures, such as meniscectomy and meniscus repair, between the two groups (p = 0.335, > 0.99). Both groups significantly improved in the anterior drawer, Lachman, pivot shift tests, and IKDC and Lysholm knee scores after surgery (all p < 0.001). The OA-ACLR group showed significantly higher rotational stability in the pivot shift test than the SB-ACLR group (p = 0.017). The postoperative side-to-side difference, the IKDC and Lysholm scores showed no significant differences between the groups (p = 0.34, 0.301, 0.438).

**Conclusions:**

OA-ACLR showed enhanced rotational stability with pivot shift test compared to SB-ACLR. It may be considered a useful alternative for revision ACL reconstruction.

## Introduction

Revision ACL reconstruction is technically more demanding than primary ACL reconstruction, and the reported outcomes of revision to date have been inferior to those of primary ACL reconstruction [1–5]. In a recent systematic review, the overall objective failure rate of revision was 13.7%, which was nearly three–four times the failure rate in a prospective series of primary reconstructions [4]. When considering revision ACL reconstruction, the surgeon must address issues such as malpositioned tunnels, bone loss, and tunnel expansion [6], all of which may influence insufficient graft fixation or strength and sometimes necessitate two-stage revision after the initial bone grafting and delay the rehabilitation program.

The over-the-top fixation ACL reconstruction technique was first introduced by MacIntosh [7]. The advantage of this technique is that the femoral fixation site for the tendon graft can be easily anticipated and that a femoral tunnel is not needed, which prevents problems caused by inappropriate tunnel position. There was also a concern regarding over-the-top fixation because of the non-isometric graft position. However, in recent biomechanical studies [8, 9], ACL reconstruction with over-the-top passage fixation has rotational stability comparable to that of ACL reconstruction with a single-bundle technique. More recent clinical studies [10, 11] have shown that equivalent clinical outcomes are anticipated in both over-the-top and traditional ACL reconstruction techniques in primary and revision settings.

Min et al. [12] introduced a modified ACL reconstruction with an over-the-top augmentation technique (OA-ACLR) and demonstrated its biomechanical superiority with enhanced rotational stability compared to single-bundle ACL reconstruction in a porcine model. By forming a sling structure over-the-top, this technique may enhance graft strength and fixation irrespective of the bone tunnel condition and enable one-stage surgery, thus presenting potential applications in revision ACL reconstruction. With several potential advantages and biomechanical superiority, we have been performing this technique in revision ACL reconstruction cases. Thus, the purpose of this study was to compare the clinical outcomes of OA-ACLR with those of single-bundle ACL reconstruction (SB-ACLR). We hypothesized that the OA-ACLR technique, which combines transcondylar ACL reconstruction with augmentation by over-the-top fixation, would enhance the postoperative knee joint rotational stability.

## Materials and methods

This retrospective comparative study compared the clinical outcomes of OA-ACLR with single-bundle ACL reconstruction (SB-ACLR).

We included 47 consecutive patients who underwent ACL revision with ACL reconstruction using the over-the-top augmentation technique from January 2003 to December 2015 at our institution. All the surgeries were performed by a single senior surgeon. The clinical outcomes of the cases were compared with those of 48 cases of primary single-bundle reconstruction with an allogenous bone-patellar tendon-bone (BTB) graft during the same consecutive period. Patients who were skeletally immature, had an intra-articular fracture, and required other ligament reconstruction surgeries, as well as those who had previous surgical procedures around the knee joint except for primary ACL reconstruction, were excluded from the study. However, patients with meniscus tears were not excluded, and treatment options, such as partial meniscectomy or meniscus repair, were performed based on the specific status of the meniscus. All patients were followed up for a minimum of 6 months.

### Surgical technique of ACL reconstruction with over-the-top augmentation technique

#### Graft preparation

**Fig. 1:**
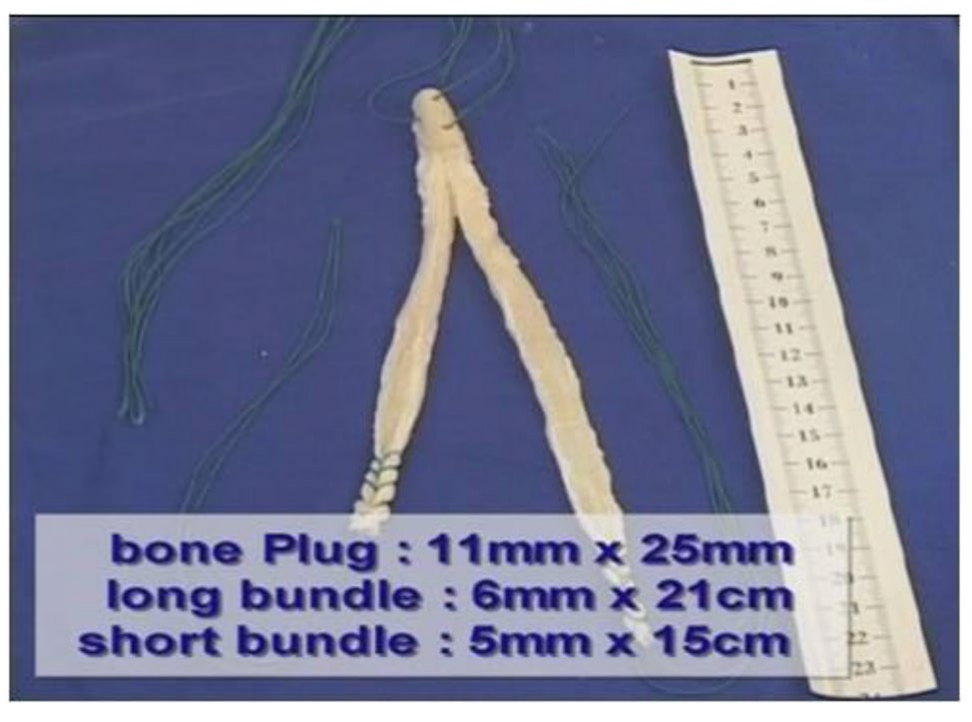
Graft preparation

An Achilles tendon allograft was used for graft preparation. The bone plug was trimmed to a cylindrical shape with a diameter of 11 mm and a length of 25 mm. The tendon was then split in half, and each of the two tendinous ends was sutured using the baseball-stitch technique with two No.5 Ethibond sutures (Ethicon, Somerville, NJ, USA), resulting in four threads retained at each end. The two bundles (long and short) of the graft were examined to determine the size of the opening they could pass through, usually 10–11 mm in diameter. Three additional No.5 nonabsorbable sutures were also passed through the bone plug to allow manual tensioning of the graft in the tibial tunnel before the final interference screw fixation (Fig. 1).

#### Tibial tunnel preparation

A tibial skin incision was made just below and medial to the tibial tuberosity for a length of 3 cm (usually along the previous skin incision scar). Using a conventional ACL tibial guide set at 55–60 °, a guide pin was inserted targeting an isometric tibial point. The tibial tunnel was then reamed sequentially using a 6 mm-, 8 mm-, 10 mm cannulated then 11 mm coring reamer. The hardware, which was inserted in primary reconstruction, was usually left in place if it did not interfere with the new tunneling procedure to minimize the residual defect. In cases of interruption by previous hardware, the metallic ones had to be removed before reaming, but the biodegradable ones were over-reamed after guidewire placement. The debris along the tunnel and intra-articular aperture were debrided using an arthroscopic shaver and a rongeur.

#### Femoral tunnel preparation

When the previous femoral tunnel was appropriately positioned, we used the previous tunnel. However, if that was not the case, we created a new tunnel as follows. The two-incision technique was used for outside-in femoral tunnel creation [13]. Briefly, an accessory lateral femoral skin incision starting at the lateral epicondyle was made proximally over the lateral distal femur, a length of 3 cm along the femur, and then the iliotibial band was split. A long Adapteur Drill Guide C-Ring (Arthrex, Naples, FL, USA) was introduced through the anterolateral portal such that the tip faced the desired isometric point of intra-articular entry for the femoral tunnel. This point is usually 7 mm anterior to the posterior edge of the medial surface of the lateral femoral condyle, resulting in the posterior cortex of the tunnel being 1 mm thick. The ACL femoral guide was set at 100–105 °and laterally rotated by 60 °from the vertical line. A Kirschner wire was then drilled through the posterolateral corner of the femoral condyle to the intra-articular entry point in an outside-in manner. After obtaining acceptable guidewire placement, the femoral tunnel was reamed sequentially up to 10 to 11 mm in diameter depending on the size of the Achilles tendon graft, which fits the summed diameter of the long and short bundles. Similarly, with the preceding tibial tunnel, any hardware on the femoral tunnel from the primary reconstruction was removed only when necessary.

#### Graft placement and fixation

Three leading suture loops were used for graft placement: two leading loops passed through both the femoral and tibial bony tunnels in opposite directions, and the other leading loop passed from the tibial tunnel, turning around the over-the-top and out to the lateral femoral skin incision. For the passage over-the-top, the two ends of the suture loop were threaded through the tip of a crochet hook suture passer. The suture passer was then introduced via the anteromedial portal, passing through the intercondylar notch over-the-top, encasing the lateral femoral condyle towards the posterolateral corner. The two ends of the suture loop were retrieved from the lateral skin incision, and the other loop end was retrieved from the exit of the tibial tunnel.

**Fig. 2:**
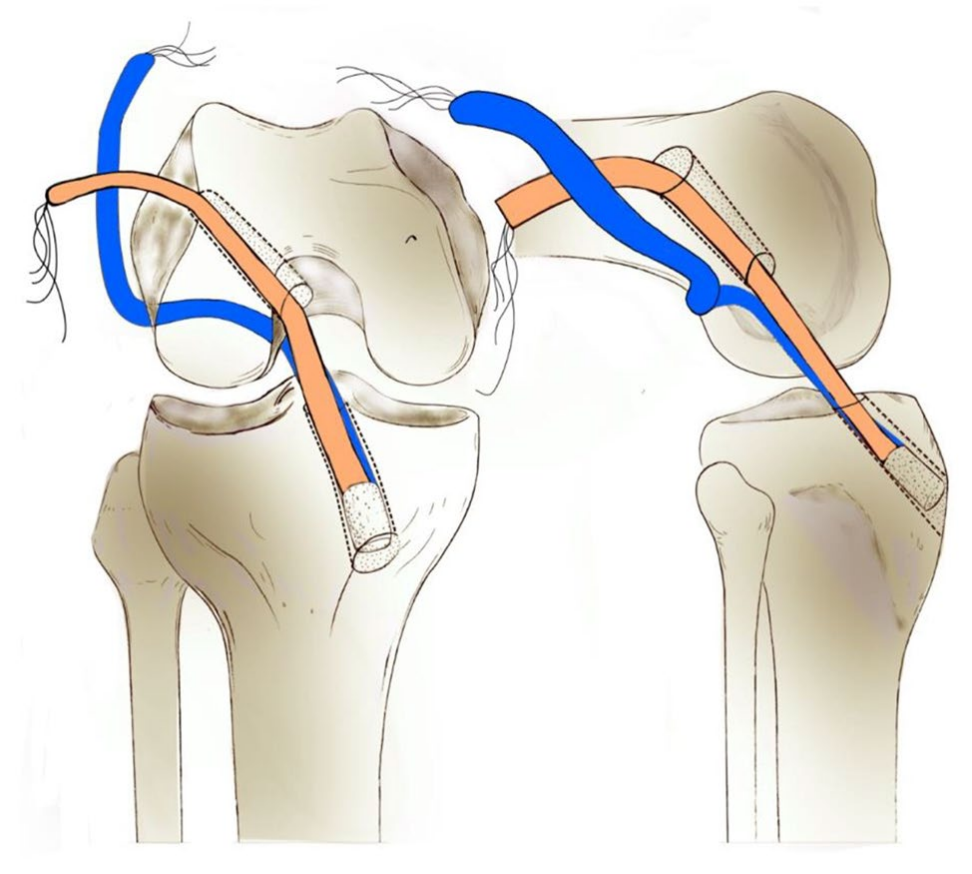
Illustration of the passage route of the graft. The short bundle (orange) passes through the femoral tunnel, and the long bundle (blue) encases the lateral femoral condyle via over-the-top

**Fig. 3:**
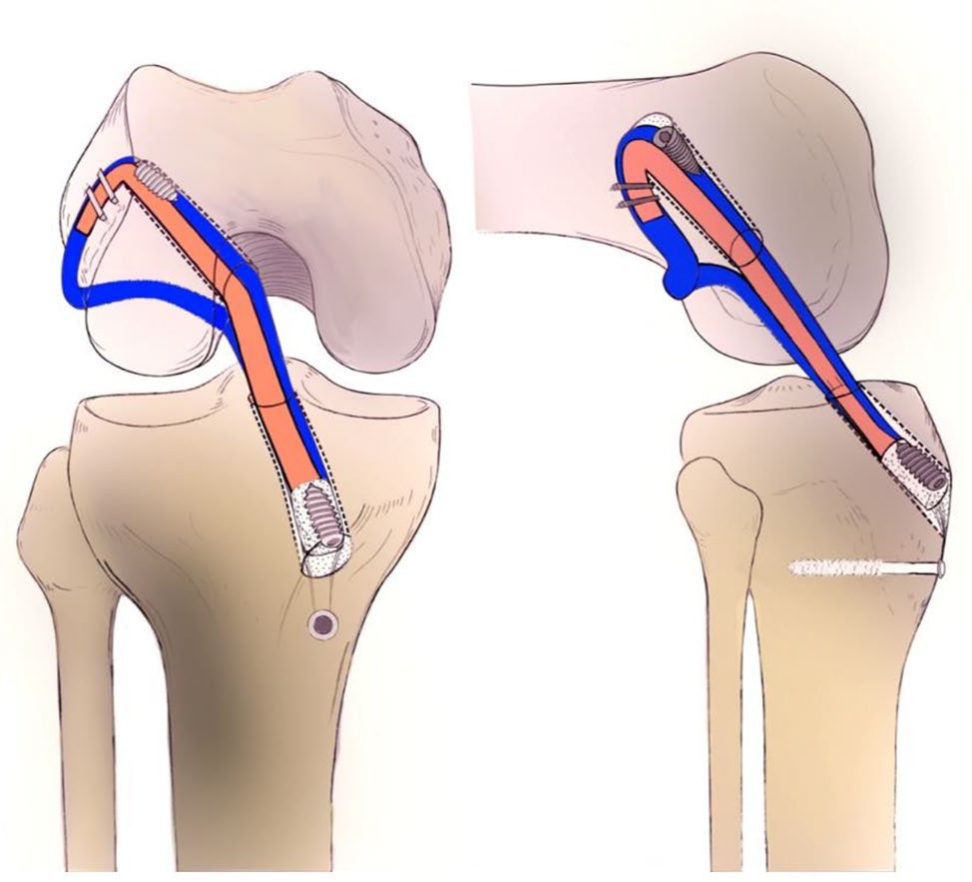
The long bundle (blue) is introduced back to the femoral tunnel outside-in forming an over-the-top sling structure of the allograft in OA-ACLR. Fixation is performed as described in the text

**Fig. 4:**
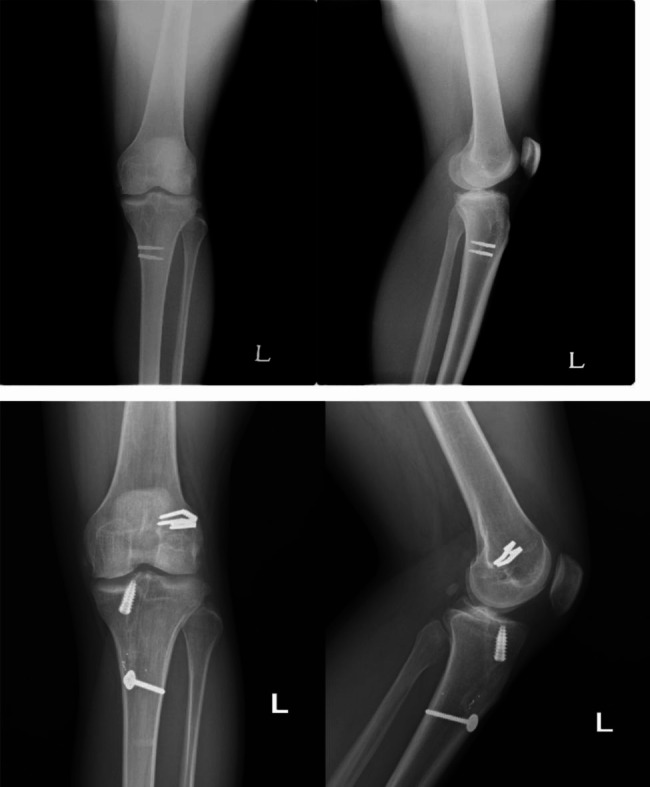
Preoperative and postoperative radiography of revision OA-ACLR

The tendinous side of the graft was first introduced through the tibial tunnel. Using a suture loop passing through both the femoral and tibial tunnels, the short bundle of the graft was passed through the tibial and femoral tunnels sequentially. Using another loop suture encompassing the lateral femoral condyle via over-the-top, the long bundle of the graft was passed through the tibial tunnel, intercondylar notch, over-the-top, and then through the lateral skin incision (Fig. 2). The long bundle was then introduced back into the joint via a femoral tunnel and returned to the tibial tunnel using another leading suture loop passing both the femoral and tibial tunnels. While simultaneously holding the residual threads from each end, the bone plug-long bundle complex was seesawed in the tibial tunnel, and the position of the bone plug was adjusted. Continuously maintaining maximum manual tension on the threads from the bone plug and the long bundle in the tibial exit, the threads from the bone plug-long bundle complex were then fixed using a washer and a cortical screw on the anteromedial tibial cortex. The end of the short bundle in the femoral exit was also subjected to an appropriate tensional force and was fixed with a bioabsorbable interference screw into the lateral femoral cortex. The remnant short bundle outside the femoral tunnel, along with the long bundle encasing the lateral femoral condyle, was double-fixed with 10 mm spike staples at the lateral femoral cortex. After confirming firm fixation on the femoral side, additional fixation was performed on the tibial side by using another interference screw (Fig. 3). Radiographs were obtained immediately after surgery (Fig. 4).

### Surgical Technique of ACL reconstruction with single-bundle technique

An allograft BTB was used for graft preparation. Each bone plug end was trimmed into a cylindrical shape with a diameter of 10 mm and a length of 25 mm. The patellar tendon was trimmed to a diameter of 10 mm. Two No.5 nonabsorbable sutures were passed through each bone plug for graft passage and manual tensioning of the graft before the final interference screw fixation.

After diagnostic arthroscopy, a tibial tunnel was created using the same method as ACL reconstruction with an over-the-top augmentation technique; however, reaming was performed up to 10 mm. For femoral tunnel preparation, a guide pin was inserted through the tibial tunnel with the isometric point 6 mm anterior to the posterior edge of the medial surface of the lateral femoral condyle as a reference point. Sequential reaming was performed up to 10 mm, resulting in the posterior cortex of the tunnel being 1 mm thick. The drilling depth was approximately 5 mm longer than the bone stock length of the BTB graft. Loop sutures were placed in the eye of the graft-passing guide pin, and the pin was pulled. Using the remaining loop-passing sutures, the trimmed allograft BTB graft was pulled into the femoral tunnel through the tibial tunnel. After proper positioning of the graft, an interference screw was inserted through the anteromedial portal. After firm fixation of the femoral side, the knee should be cycled through a full range of motion to confirm graft placement and adjust the graft before tibial fixation. The knee was placed at approximately 10˚-20˚ of flexion, and the tibial side was fixed using an interference screw.

### Evaluation of clinical outcomes

The anterior drawer test, Lachman test, and pivot shift test were performed preoperatively and at the final follow-up to evaluate knee instability. All the physical examinations were performed by a experienced single senior surgeon. The anterior drawer test was conducted at 90 degrees of knee flexion, while the Lachman test was performed at 20–30 degrees of flexion. The anterior translation was classified based on the International Knee Document Committee criteria, with normal as up to 2 mm, grade 1 as 3 to 5 mm, grade 2 as 6 to 10 mm, and grade 3 as more than 10 mm. For the pivot shift test, a grade of 1 + was assigned if a subtle change of motion or glide without an appreciable articular clunk was present. A 2 + pivot shift indicated a distinct clunk, while a grade of 3 + represented a gross clunk or locking in the subluxated position [14]. An anterior stress radiograph was obtained by applying 150 N anterior stress in a 90-degree knee flexion state using a Telometer (Daiseung Medics, Seoul, South Korea). Side-to-side differences were compared with the non-affected side at the final follow-up. Functional evaluation was performed using the IKDC subjective and Lysholm knee scores before surgery and at the final follow-up.

### Statistical analysis

The data were analyzed using the SPSS statistical package (SPSS 25, Chicago, Illinois). For continuous variables, the *t*-test was used to compare the two groups. For non-continuous variables, either the chi-squared test or Fisher’s exact test was performed. The level of significance was set at p < 0.05.

## Results

The patients’ demographic data are summarized in Table 1. There was no difference between the two groups in terms of sex, age, BMI, and etiology. Concomitant meniscectomy was performed on 16 patients in the OA-ACLR group and 22 patients in the SB-ACLR group. Additionally, meniscus repair was performed on 4 and 5 patients in the OA-ACLR and SB-ACLR groups, respectively. The statistical analysis revealed no significant differences concerning concomitant meniscal surgical procedures between the two groups (p = 0.335, > 0.99). Preoperative and final follow-up evaluations of each group showed significant improvement in the anterior drawer, Lachman, and pivot shift tests (all p < 0.001). All patients were followed up for a minimum of 6 months, and the follow-up period revealed no significant differences between the two groups (15.4 ± 12.8 vs. 12.3 ± 7.4, p = 0.156). At the final follow-up, there was no significant difference in the anterior drawer and Lachman tests between the two groups (p = 0.787, 0.305, respectively). The OA-ACLR group showed significantly higher rotational stability in the pivot shift test than in the SB-ACLR group (p = 0.017). The results are presented in Table 2.

**Table 1:** Demographic data

	Over-the-top augmentation (n = 47)	Single bundle reconstruction (n = 48)	p-value
**Graft type**	Allogenous Achilles bone tendon	Allogenous bone-patellar tendon-bone	
**Gender (M:F)**	40 : 7	43 : 5	0.796
**Age (years)**	30.9 ± 10.8	33.0 ± 10.6	0.350
**BMI (kg/m** ^**2**^ **)**	25.1 ± 4.0	24.4 ± 2.7	0.355
**Concomitant surgical treatment**			
Meniscectomy (%)	16 (34.0%)	22 (45.8%)	0.335
Meniscus repair (%)	4 (8.5%)	5 (10.4%)	> 0.99
Etiology			0.324
Sports	35	35	
Fall down	4	7	
Traffic accident	6	2	
Unknown	2	4	
**Follow-up (Months)**	15.4 ± 12.8	12.3 ± 7.4	0.156

BMI; body mass index

**Table 2:** Instability test

	Over-the-top augmentation (n = 47)		Single bundle reconstruction (n = 48)		p-value
	Pre-op	Final f/u	Pre-op	Final f/u	
Anterior Drawer test	N(3)	N(42)	N(8)	N(43)	p = 0.787
	I(10)	I(3)	I(11)	I(4)	
	II(21)	II(2)	II(16)	II(1)	
	III(13)	III(0)	III(13)	III(0)	
p-value	**p < 0.001**		**p < 0.001**		
Lachman test	N(0)	N(37)	N(0)	N(44)	p = 0.305
	I(14)	I(8)	I(14)	I(1)	
	II(15)	II(2)	II(18)	II(3)	
	III(18)	III(0)	III(16)	III(0)	
p-value	**p < 0.001**		**p < 0.001**		
Pivot shift test	N(0)	N(46)	N(0)	N(40)	**p = 0.017**
	I(21)	I(1)	I(15)	I(7)	
	II(15)	II(0)	II(18)	II(1)	
	III(11)	III(0)	III(15)	III(0)	
p-value	**p < 0.001**		**p < 0.001**		

N; normal, I; grade 1, II; grade 2, III; grade 3

In functional evaluation, the IKDC and Lysholm knee scores showed significant postoperative improvement in both groups, and the values are shown in Table 3 (both p < 0.001). There was no significant difference in the postoperative IKDC and Lysholm scores between the two groups (p = 0.301, 0.438, respectively). The side-to-side difference measured at the final follow-up was 4.1 mm for OA-ACLR and 3.6 mm for SB-ACLR and there was no significant difference between the two groups (p = 0.34).

**Table 3:** Subjective Functional Scoring

	Over-the-top augmentation (n = 47)	Single bundle reconstruction (n = 48)	p-value
Preoperative IKDC	60.7 ± 5.3	59.9 ± 5.4	0.461
Postoperative IKDC	83.2 ± 11.0	79.0 ± 8.4	0.301
p-value	**p < 0.001**	**p < 0.001**	
Preoperative Lysholm	61.6 ± 4.7	62.2 ± 4.7	0.511
Postoperative Lysholm	85.7 ± 9.1	88.8 ± 10.4	0.438
p-value	**p < 0.001**	**p < 0.001**	

## Discussion

Our key findings are that the modified ACL reconstruction with an over-the-top augmentation technique in revision surgery showed enhanced rotational stability and comparable clinical outcomes to primary SB isometric ACL reconstruction with an allogenous BTB graft.

ACL revision is known to be similar in stability to primary ACL reconstruction, but worse in terms of patient-reported outcomes and failure rates [1, 4, 5, 15]. Among the various causes of ACL revision, ACL tunnel malpositioning has received the most attention, and other tunnel-related complications, including widening, loss of containment, and hardware interference are also common [16]. If there is no critical tunnel widening, overlap, etc., single-stage revision is possible; otherwise, a second-stage revision is required after the bone graft.

When the over-the-top technique is used in ACL revision, single-stage revision is possible, regardless of the state of the femoral tunnel. Over-the-top fixation was first introduced by MacIntosh, and modifications, such as additional lateral plasty [17] or non-anatomic double bundle reconstruction [18] have been attempted. In biomechanical studies [8, 9], over-the-top fixation ACL reconstruction showed similar or better stability to single-bundle anatomical ACL reconstruction, and clinical results showed comparable outcomes in primary or revision settings [10, 11, 19]. In addition, over-the-top fixation has been verified for a long-term outcome of more than 10 years, it has also been considered comparable to other techniques [20–23]. We designed a surgical technique that considers robust fixation with thicker bundles, combining single-bundle ACL reconstruction and over-top fixation, which was the first clinical result of this technique and showed improved rotational stability over primary single-bundle ACL reconstruction.

This technique, which is primarily based on the over-the-top technique, particularly resembled the non-anatomic double bundle reconstruction method [18]. The non-anatomical double bundle reconstruction method has been shown biomechanically comparable or even more stable when compared to anatomical double bundle reconstruction [10, 23]. It has exhibited superior outcomes in long-term randomized studies spanning a minimum of 8 years when contrasted with single bundle reconstruction [24]. Furthermore, even when utilizing allografts, the average success rate over a period of approximately 6 years is approximately 81%, which is similar to other ACL revision results [4, 25]. Our over-the-top augmentation technique differs by allowing the long bundle of the over-the-top approach to re-enter the femoral tunnel and pass through to the tibia tunnel, thus enabling sufficient graft thickness in both the femur and tibia tunnels.

Several advantages can be addressed regarding our over-the-top augmentation technique for revision ACL reconstruction. The greatest advantage may be that a one-stage revision can be accomplished regardless of the tunnel conditions. Utilizing a 6 mm thick long bundle, a 5 mm thick short bundle, and an additional interference screw for the femoral tunnel, along with an 11 mm thick bone plug, a 6 mm thick long bundle, and additional interference screws for the tibial tunnel, all secured within a single tunnel, ensures robust fixation even in the presence of tunnel widening. Furthermore, this enhanced stability could potentially enable a more active rehabilitation program. In this study, we allowed all patients full weight-bearing ambulation as well as full range-of-motion exercises immediately after surgery. No ACL brace was necessary for protective purposes. Based on the strong confidence in the graft strength and fixation by our over-the-top augmentation technique, facilitated rehabilitation is feasible in revision ACL reconstruction.

In OA-ACLR, this firm and stable graft fixation can promote rehabilitation, so it is thought that there was no difference in subjective patient outcomes in this study compared with SB-ACLR. In addition, although there was no difference in the anterior draw and Lachman tests, rotational stability was better than that of SB-ACLR in the pivot shift test. In some meta-analyses, revision ACL reconstruction showed no significant difference in instability compared with primary reconstruction; however, in this study, OA-ACLR showed statistically better results in rotational stability [5]. Theoretically, rotational instability is caused by abnormal lateral translation of the knee joint [26]. Thus, the over-the-top sling structure in the tensioned allograft anchored in the lateral femoral condyle may grasp and hold it in situ against the tibia and potentially suppress the abnormal lateral translation of the knee joint. Min et al. [12] demonstrated that over-the-top sling structures inhibited lateral translation in a porcine model with OA-ACLR.

The limitation of our clinical study is that it was not a prospective randomized controlled study and included different patient groups from the primary and revision settings. We did not use other revision techniques in the same period and compared the clinical results of the over-the-top augmentation technique with those of the primary ACL reconstruction performed consecutively during the same period. Our institution is a tertiary center, and patients with failed ACL reconstruction are often referred to our institution. We chose patients who underwent primary ACL reconstruction with allogenous BTB as a control group because both groups utilize allogenous grafts and allow bone-to-bone healing, and these conditions create the most biochemically similar environment between the two groups. In addition, since primary ACL reconstruction is generally reported to show at least comparable or better clinical results than revision ACL reconstruction, we thought that it would be meaningful to compare it with the control group, which is generally expected to have better clinical results. In terms of different tendon grafts of Achilles-bone-tendon and bone-patellar tendon-bone, Ahn et al. [1] reported that the difference in allogenous tendons does not affect the results, so the impact of this on the outcome would be minimal.

In addition, it is difficult to evaluate long-term outcomes due to the short follow-up period, and another limitation is that complications such as graft failure, meniscal injury, and cartilage injury that may occur after surgery may not have been evaluated.

Also, regarding general over-the-top techniques, while there have been numerous studies conducted, our technique is limited by the availability of only biomechanical data, without the presence of comparative studies with other over-the-top techniques. Nevertheless, our study has the strength that revision ACL reconstruction with the over-the-top augmentation technique showed similar clinical results to primary ACL reconstruction, even if the follow-up period was short, and showed better rotational instability. A randomized, well-designed prospective study comparing the revision techniques could offer more substantial evidence.

## Conclusion

OA-ACLR showed enhanced rotational stability with pivot shift test compared to SB-ACLR. It may be considered a useful alternative for revision ACL reconstruction.

## Data Availability

The datasets used and/or analysed during the current study available from the corresponding author on reasonable request.
